# How Different Preparation Techniques Affect MRI-Induced Anxiety of MRI Patients: A Preliminary Study

**DOI:** 10.3390/brainsci13030416

**Published:** 2023-02-27

**Authors:** Zuhal Y. Hamd, Amal I. Alorainy, Lena A. Alrujaee, Maha Y. Alshdayed, Afrah M. Wdaani, Atheer S. Alsubaie, Layal A. Binjardan, Sarab S. Kariri, Rawan A. Alaskari, Marah M. Alsaeed, Mohammed Awad Alharbi, Marzouk Sari. Alotaibi, Nagwan Elhussein, Mayeen Uddin Khandaker

**Affiliations:** 1 Department of Radiological Sciences, College of Health and Rehabilitation Sciences, Princess Nourah bint Abdulrahman University, Riyadh 11671, Saudi Arabia; 2 Medical Imaging Department, King Abdullah bin Abdulaziz University Hospital, Princess Nourah bint Abdulrahman University, Riyadh 11671, Saudi Arabia; 3 Department of Diagnostic Radiology, College of Applied Medical Sciences, University of Ha’il, Hail 53962, Saudi Arabia; 4 Centre for Applied Physics and Radiation Technologies, School of Engineering and Technology, Sunway University, Bandar Sunway 47500, Selangor, Malaysia; 5 Department of General Educational Development, Faculty of Science and Information Technology, Daffodil International University, DIU Rd, Dhaka 1341, Bangladesh

**Keywords:** magnetic resonance imaging, anxiety, State-Trait Anxiety Inventory (STAI), routine preparation, video preparation

## Abstract

Background: Magnetic resonance imaging (MRI) exams may cause patients to feel anxious before or during the scan, which affects the scanning outcome and leads to motion artifacts. Adequate preparation can effectively alleviate patients’ anxiety before the scan. We aimed to assess the effect of different preparation methods on MRI-induced anxiety: We conducted a prospective randomized study on MRI patients between March and May 2022. We divided 30 patients into two groups: the control group, which received routine preparation (RP), and the experimental group, which received video preparation (VP). We used the State-Trait Anxiety Inventory (STAI) to measure anxiety levels before and after the interventions. We assessed patients’ self-satisfaction after the scan: After preparation, VP (STAI mean = 10.7500) and RP (STAI mean = 12.7857), we observed a significant association between the pre- and post-STAI results in VP (*p* = 0.025). The effects of both methods in decreasing anxiety were more significant for first-timers (*p* = 0.009 in RP/0.014 in VP). We noted high satisfaction levels for both forms of preparation. The VP technique was superior in reducing patient anxiety, especially in first-time MRI patients. Hence, VP techniques can be used in different clinical settings to reduce anxiety and facilitate patients’ understanding of the instructions given.

## 1. Introduction

Since the 1980s, there has been a significant rise in magnetic resonance imaging (MRI), which has become one of the leading modalities used in the clinical field. MRI has evolved as one of the most important diagnostic imaging techniques. In addition, MRI is the modality of choice for imaging abnormalities due to its superior illustration and contrast of soft tissues [1,2]. It is also thought to be biologically safe and pain free [3], in contrast to computed tomography (CT) and X-ray procedures [4,5]. MRI uses a high-energy magnetic field with radio waves to produce detailed images of the human body with no radiation hazards [1]. In pregnancy, MRI is recommendable, and there is no evidence that MRI can damage the fetus [6,7]. Unfortunately, numerous patients experience a certain level of anxiety before or during the procedure due to the nature of the exam [8]. Barlow defined anxiety as a “negative mood state that is accompanied by bodily symptoms such as increased heart rate, muscle tension, a sense of unease, and apprehension about the future” [9]. Up to 37% of MRI patients suffer from moderate to high anxiety [10]. The narrow scanner, noisy sequences, lengthy scan, restricted movement, fear of pain, and expectations of exam results all contribute to patients’ anxiety [2,3].

The slightest movement will deteriorate image quality and affect diagnostic accuracy. Thus, patient immobilization during the entire procedure is necessary to achieve high-quality images [11]. Anxiety during MRI leads to an increased respiratory rate, bowel movements, and fluid flow, all of which could distort image quality [10]. A previous study estimated that 11.2% of patients did not show up for their appointment times [11]. Patient anxiety can lead to patient movement, which consumes cognitive effort and reduces the patient’s ability to stay still, requiring the MRI examination to be repeated; as a result, the examination time increases and expensive resources are consumed [12,13]. Moreover, patients tend to terminate their exam before it ends or be absent due to their anxiety. Anxious patients have early termination rates ranging from 1.22% to 39%, which will have an impact on the patient and health care institutions because they will be affected financially due to incomplete scans or patients’ absence [1].

Anxiety is considered a reason for motion artifact and the early termination of exams, eventually affecting the scanning outcome. Thus, researchers seek to examine various strategies to reduce it. Mock MRI, sedation, and cognitive behavioral therapy are useful options to prevent anxiety. However, most of them are time- and cost-consuming, demanding, or do not apply to all patients’ informational needs [10]. Multiple techniques for reducing anxiety during MRI have been proposed [14,15]. Audio-guided self-hypnosis is one such method. A recent study found that audio-guided self-hypnosis techniques significantly reduced claustrophobia in high-risk MRI patients [16]. In addition, previous research has advocated for staff to receive comfort talk training, as it teaches MRI personnel how to effectively establish mutual understanding with patients and guide them away from uncomfortable thoughts. However, the success of comfort talk training is highly subjective and dependent on personnel responses [12].

Adequate preparation by staff is one of the chief purported methods of alleviating patients’ stress [1]. Written brochures and visual videos could assist patients before the scan [17]. Given its audio and visual content, multiple studies have found video-based information to be superior compared to written information [17]. Visual preparation promotes patient satisfaction and reduces anxiety before various clinical procedures [10].

Although several studies conducted worldwide have shown results consistent with our hypothesis, claiming that sufficient preparation influences patients’ anxiety levels, the topic cannot be generalized broadly due to geographic and social differences [10]. Therefore, to validate the findings of this specific topic and to expand the scope of research at different levels, we performed an experiment on 30 MRI patients at King Abdullah bin Abdul-Aziz University Hospital (KAAUH) in Riyadh, Saudi Arabia to evaluate the effectiveness of additional visual preparation in reducing anxiety in patients undergoing MRI.

Our study aimed to measure and analyze the differences in anxiety level before and after routine and video preparation of patients undergoing Magnetic Resonance Imaging. In addition, we compared the effectiveness of different preparation methods on anxiety level of first time and non-first time MRI patients and finally assessed patients’ satisfaction regarding different types of preparations.

## 2. Methods and Materials

We conducted a prospective, randomized control trial with male and female patients in the MRI department at KAAUH in Riyadh, Saudi Arabia. The 30 patients ranged in age from 18 to 60 years and spoke Arabic and English. We excluded patients who were unable to give informed consent, patients with anxiety disorders, sedated patients, inpatients, and those undergoing feet-first exams.

We measured patients’ anxiety using the State-Trait Anxiety Inventory (STAI) to assess the effect of different preparation methods [18], after receiving approval from the institutional review board (IRB No: 22-0037) of Princess Nourah bint Abdulrahman University, as well as from KAAUH (IRB No: 1-70004-024), during the period from 28 March to 13 May 2022.

### 2.1. Research Methods

Using the STAI, we assessed the effects of different preparation methods on the level of anxiety in patients who were willing to undergo an MRI exam. The STAI, developed by Spielberger in 1983, consists of 40 questions measured on a 4-point Likert scale and is commonly employed in the clinical field to gauge trait and state anxiety [19]. We randomly selected two groups based on odd and even numbered days. After patients arrived at the radiology department’s main lounge, we instructed both groups to fill out a soft copy of the (pre-intervention) STAI. We asked the control group patients, who were scheduled for an MRI exam on odd numbered days, to undergo the hospital’s routine preparation (RP) (signing the informed consent form for the MRI exam, as well as regular preparation with a technologist). We asked the experimental group patients, who were scheduled for an MRI exam on even numbered days, to watch a YouTube video on MRI made by us using quick response (QR). After the interventions, we guided both groups in filling out a soft copy of the (post-intervention) STAI. We then measured the results of the two tests (pre- and post-STAI) for each group and correlated the outcomes with each other. We used a valid Arabic translation of the original STAI for Arabic speakers [18]. To reduce patient dropout, prevent disturbances in the dynamics of the department, and save time, we used a valid and acceptable short form of the STAI developed in 1992 [20]; it contains the 6 core questions (the full version has 40 questions) and provides concise and acceptable measurements compared to the longer version [20]. As shown in Table 1, after the exam, we asked both groups to fill out a self-questionnaire to assess their satisfaction with RP and VP, respectively. 

This short version of the STAI involves six questions that the patient should answer. According to the answers (note that questions 1, 4, and 5 had flipped scores), we calculated the score. The score for the highest anxiety level was 24, while the lowest score was 6.

### 2.2. Statistical Analysis

We collected the data and tabulated them using the Statistical Package for the Social Sciences (SPSS), Version 26, (IBM, SPSS Inc. IBM Corp. Chicago, USA) to assess the impact of the different preparation methods. We employed a paired *t*-test to compare the outcomes between the pre- and post-intervention tests, specifically the mean within one group where the level of significance was set at *p* < 0.05. We also performed an independent samples *t*-test to compare the mean anxiety between the two groups, along with patient satisfaction, where the level of significance was set at *p* < 0.05. 

### 2.3. Ethical Considerations

We conducted the study at KAAUH. We obtained approval from the institutional review board (IRB No: 22-0037) of Princess Nourah bint Abdulrahman University, as well as from KAAUH (IRB No: 1-70004-024). Participation was voluntary without compensation. The participants were informed about the study’s objectives and their rights, including the right to withdraw at any time. Prior to the study, each participant signed an informed consent form, and was told that all identifying information would be kept confidential and used only for the purposes of the study.

## 3. Results

In total, 30 patients participated; 14 were in the control group and 16 were in the experimental group, VP (Table 2 and Table 3).

However, the majority of participants were female (81.3% in the VP group and 35.7% in RP group), as seen in Table 2 and Table 3. Accordingly, this study represents the female perspective more than the male one. Moreover, most of the participants were between 50 and 60 years old, as indicated in Table 4 and Table 5.

Another goal of this study was to compare the effectiveness of different preparation methods (RP and VP) on anxiety levels between first-time and non-first-time MRI patients. Table 6 indicates that 25% of the VP group consisted of first-timers, whereas 75% were non-first timers. Furthermore, Table 7 revealed that 35.7% of the RP group were first-timers and 64.3% were non-first-timers.

As indicated in the table, since the *p*-value was less than 0.05, we concluded that there was an association between the pre- and post-STAI results in the VP group (*p* < 0.05). However, there was no association between the pre- and post-STAI results in the RP group (*p* > 0.05) because the *p*-value was greater than our chosen significance level (*p* = 0.099).

## 4. Discussion

Additional VP reduces patient anxiety before an MRI scan [2,10]. With this study, we aimed to assess the effects of different preparation methods on the anxiety levels of patients undergoing MRI exams at KAAUH by measuring and analyzing the differences in anxiety levels before and after RP and VP using the STAI. The questions were normally distributed. We also randomized the participants; there was no bias regarding age or gender for any group. 

The average STAI outcomes of the 14 MRI patients who underwent RP and the 16 patients who received VP are outlined in Table 8, which indicates that before preparation, 14.92 ± 4.26 was the mean and SD of the STAI score for the RP group and 14.68 ± 3.02 for the VP group. Inadequate preparation is one of the main causes of anxiety before a scan [1]. Moreover, previous research has been conducted to assess the factors that contribute to patients’ anxiety during MRI scans; 42% of patients stated that a lack of specific information regarding the exam and its nature is the most common cause of anxiety [14]. Additionally, the maximum STAI score before both types of preparation, as seen in Table 8, was 23 for the RP group and 22 for the VP group; these scores are considered to be high. If anxious patients undergo an MRI exam, this may result in motion artifacts, which could influence the scanning outcome; this outcome is similar to previous studies [2,14].

Following preparation, both groups filled out another STAI for a comparison of the influence of the two preparation methods. As demonstrated in Table 8, 12.78 ± 2.93 was the mean and SD of the post-STAI score for the RP group and 10.75 ± 3.15 for the VP group. The RP group experienced a slight change in anxiety level as the STAI score declined from 14.92 to 12.78. Statistically, there was no association between the STAI score before and after RP, since the level of significance was *p* > 0.05, as outlined in Table 9. Conversely, as listed in Table 8, the average anxiety score of the 16 patients who received VP revealed a significant reduction in anxiety level (14.68) before preparation and (10.75) after preparation. In addition, according to Table 9, there was a correlation between the pre- and post-STAI scores in the VP group, as the significance level was *p* < 0.05. Further, Table 8 shows that the maximum STAI score in both groups after preparation was 18 for the RP group and 17 for the VP group. These results indicate that both techniques are useful in decreasing high anxiety level.

The findings of Table 8 and Table 9 support the outcomes of past studies [8,17], which imply that written and verbal instructions have a minor effect on reducing anxiety levels prior to an MRI exam compared to VP. Moreover, traditional written and verbal preparation does not contain sufficient information for patients to become fully aware of what will happen during an MRI scan, such as the scanner’s shape or an example of MRI-related noise, which are factors that may lower patients’ MRI-induced anxiety [3,15]. Further, VP is an easy, simple technique that can be used with all patients prior to an MRI exam with minimum costs while providing better information about the exam. Thus, patients will be less anxious and more cooperative during the exam. Overall, we need to ensure that patient anxiety is reduced before and during an MRI exam to prevent consequences that may influence the outcome, such as altered image quality and early exam terminations [1,12,15]. 

One objective of our study was to compare the effectiveness of different preparation methods (VP and RP) on anxiety levels between first-timers and non-first-timers. As Table 9 demonstrates, the mean STAI score of both groups decreased after preparation. The mean STAI score of first-timers in the VP group dropped from 15.6 to 11.2, whereas that of first-timers in the RP group fell from 17.8 to 15.2. Certainly, adding VP significantly reduced anxiety level in first-timer patients; patients who attend their first MRI exam and do not have prior knowledge of what they will be experiencing are likely to be more anxious. In this regard, since anxiety is often increased by insufficient knowledge about an MRI exam, any additional information that can be provided before the scan (in whatever form, including VP, as we proposed) should positively impact the patient’s anxiety level.

Following previous studies [14,15], involving similar experiments but only with MRI first-timers, we aimed to expand knowledge on the effectiveness of different preparation methods to include also non-first-timer MRI patients. Table 10 indicates that the average STAI score of the VP group of non-first-timers declined from 14.8 to 11.3, which implies that VP can also decrease MRI-induced anxiety in this group, although the reduction is not as significant as with first-timer patients. Likewise, the average STAI score in the RP group fell from 13.3 to 11.4. According to these results, there was little or no difference between the anxiety level of the VP and RP groups, since they had already received most of the information mentioned via video, written, or verbal form.

**Figure 1:**
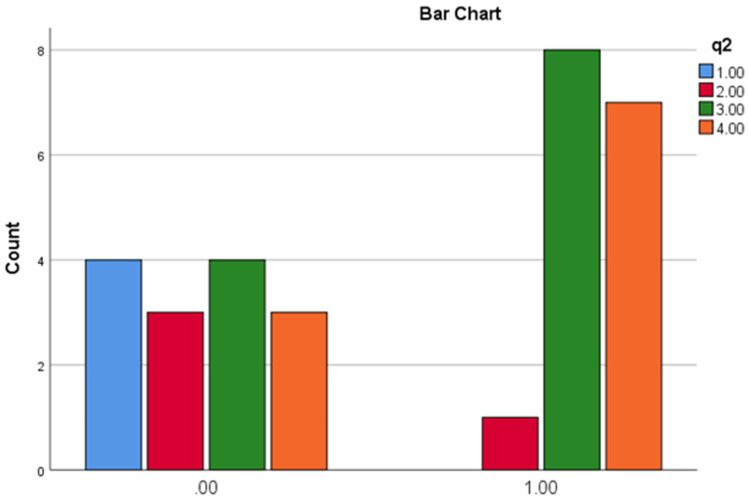
Patient self-satisfaction results for question 7: Did the preparation for MRI prior to the scan alleviate your anxiety?

**Figure 2:**
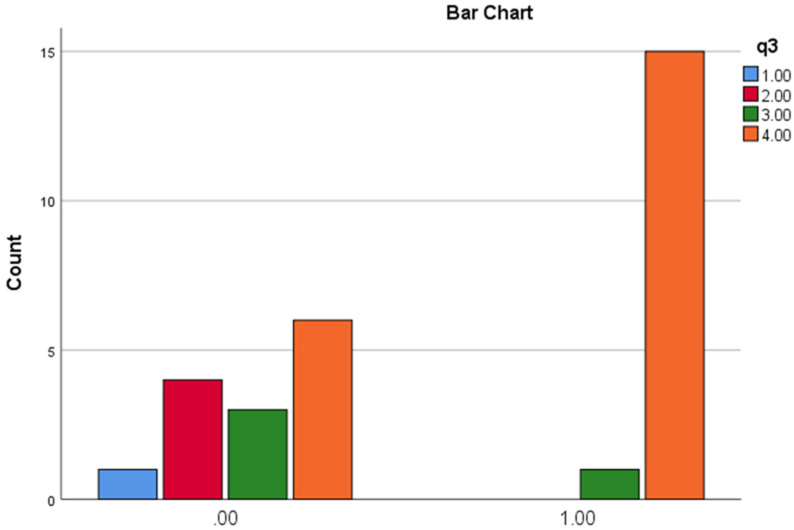
Patient self-satisfaction results for question 8: Did you feel that the information you received prior to the scan corresponded comprehensively to what actually happened during the scan?

After the exam, both groups were administered a questionnaire with four questions to assess satisfaction regarding the preparation methods and their MRI experience. We observed two especially remarkable findings. The bar chart in Figure 1 shows the answers to Question 7 (Did preparing for the MRI exam prior to the scan alleviate your anxiety?). As for the RP group, 4 out of 14 participants marked no, 3 out of 14 chose slightly, 4 out of 14 selected sort of, and 3 out of 14 answered a lot. In the VP group, 1 out of 16 participants chose slightly, 8 out of 16 selected sort of, and 7 out of 16 answered a lot. Furthermore, Figure 2 displays the answers to Question 8 (Do you feel that the information you received prior to the scan was comprehensive compared to what actually happened during the scan?). In the RP group, 1 out of 14 participants answered no, 4 out of 14 chose slightly, 3 out of 14 picked sort of, and 6 out of 14 stated a lot. Conversely, in the VP group, 1 out of 16 participants selected sort of, and 15 out of 16 stated a lot. Table 11 shows the correlations between the different types of preparation (VP and RP) and patient satisfaction. Statistically, there was a significant correlation between both types and patient satisfaction (*p* = 0.009/0.014, respectively). According to the responses, both methods were satisfactory, but patients who received VP were slightly more satisfied than those who received RP as mentioned by [20,21].

As indicated in Table 11, since the *p*-value was *p* = 0.009 and 0.014 for RP and VP, respectively, we concluded that there was an association between both types of preparation (VP and RP) and patient satisfaction (*p* < 0.05).

Possible answers included no (blue), slightly (red), sort of (green) and a lot (orange) (0.00 for RP and 1.00 for VP).

Possible answers included no (blue), slightly (red), sort of (green) and a lot (orange) (0.00 for RP and 1.00 for VP).

Our overall results support the outcomes of prior studies [10,15,17], which found that VP is a superior technique that can alleviate MRI-induced anxiety. In addition, VP is an easy, cost-effective method that can be applied in different hospital departments and clinical settings to facilitate patients’ understanding of instructions. Thus, patients will be more cooperative during exams, which in turn will save staff members time and effort. Verbal and written instructions are helpful to patients and can be an additional source of information in conjunction with videos where patients’ questions can be answered.

Generalization of the results needs to be investigated further. We had a small sample size, primarily due to time restrictions; the effects could be better understood with a larger sample size. Intervention techniques can affect the outcomes; a previous study compared cortisol and prolactin levels using blood samples from patients before and after preparation [3]. We did not use this intervention in our research; instead, we implemented one method only: the STAl. In another study, two interventions were harnessed for comparison: a short video, and a telephone conversation with the radiographer [10]. In our study, we involved one intervention due to time limits. Such interventions increase the accuracy of the findings and support the results. Another limitation was the exclusion of certain patients from the sample. Including other patient groups such as inpatients, those receiving feet-first exams, and younger age groups would significantly improve the generalizability of the findings. In addition, each patient’s referral indication was unknown. This affected the investigation because the leading cause of pre-scan anxiety is influenced by the purpose of the exam [2,3]; hence, it is reasonable to assume that a referral indication such as cancer would increase patients’ anxiety. However, the questionnaire has high validity and reliability which were tested first.

## 5. Conclusions

This study demonstrates the effectiveness of two different preparation techniques in reducing patient anxiety before an MRI exam. We included a comparison between routine (verbal and written) preparation and a video that showed patients what to expect from the exam. In line with the findings of previous studies [16,19], both techniques reduced patients’ anxiety levels, with VP demonstrating a significant reduction in patient anxiety, especially for first-timers. The video link is easy to apply and can be shared with different patients before their MRI scans in very little time. VP can also be implemented routinely and replace face-to-face interactions with the radiographer. A 2-min video in a crowded waiting room could be helpful for the facility and staff. By contrast, a routine conversation with a radiographer may be more challenging due to time constraints. This could be an additional source of information, in conjunction with the video where patients’ questions can be answered.

## 6. Strengths and Limitations

The current study has several strengths. The visual preparation technique demonstrated significant reduction in patient anxiety in comparison to other preparation techniques. This visual information can be clearly understood by different individuals and also the video link is easy to apply and can be implemented routinely prior to different clinical examinations. In contrast, there are some limitations to our findings; the small sample size, exclusion of inpatient and feet-first exams and restricted data collection period.

## 7. Recommendations

Video Preparation (VP) was effective at alleviating various degrees of patient anxiety in our experiment. VP is easy to implement in multiple areas and departments, regardless of social or geographic differences. We recommend using VP in every MR department to reduce anxiety and to make it easier for patients to understand instructions. We also suggest applying the experiment to a broader range of the population and that future studies include bigger samples and diverse cases, with no exclusions.

## Figures and Tables

**Table 1:** The 6-items STAI.

Question No	Statement	Not at All	Somewhat	Moderately	Very Much
1-	I feel calm	1	2	3	4
2-	I am tense	1	2	3	4
3-	I feel upset	1	2	3	4
4-	I am relaxed	1	2	3	4
5-	I feel content	1	2	3	4
6-	I am worried	1	2	3	4

**Table 2:** Frequency of gender distribution among patients who received VP (Video Preparation) (the study group).

VP: Video preparation	Females	13	81.3
	Males	3	18.8
Valid responses	Total	16	100.0

**Table 3:** Frequency of gender distribution among patients who received Routine Preparation (RP) (the control group).

RP		Frequency	Percent
Valid responses	Male	9	64.3
	Female	5	35.7
	Total	14	100.0

**Table 4:** Frequency of age distribution among patients who received VP (the study group).

VP	Age	Frequency	Percentage
Valid responses	20–29	1	6.3
	30–39	2	12.5
	40–49	5	31.3
	50–59	8	50.0
	Total	16	100.0

**Table 5:** Frequency of age group distribution among patients who received RP (the control group).

RP	Age	Frequency	Percentage
Valid responses	20–29	4	28.6
	30–39	3	21.4
	40–49	3	21.4
	50–60	4	28.6
	Total	14	100.0

**Table 6:** Frequency of first-timers and non-first-timers regarding MRI exam distribution among patients who received VP (the study group).

VP		Frequency	Percentage
Valid responses	First	4	25.0
	Non-first	12	75.0
	Total	16	100.0

**Table 7:** Frequency of first-timers and non-first-timers regarding MRI exam distribution among patients who received RP (the control group).

RP		Frequency	Percent
Valid responses	First	5	35.7
	Non-first	9	64.3
	Total	14	100.0

**Table 8:** Descriptive statistics of the total STAI scores pre-/post-RP and pre-/post-VP.

	N	Minimum	Maximum		Mean	Std. Deviation
	Statistic	Statistic	Statistic	Statistic	Std. Error	Statistic
Pre-RP STAI	14	8.00	23.00	14.9286	1.14097	4.26911
Post-RP STAI	14	8.00	18.00	12.7857	0.78571	2.93987
Pre-VP STAI	16	11.00	22.00	14.6875	0.75674	3.02696
Post-VP STAI	16	6.00	17.00	10.7500	0.78793	3.15172

**Table 9:** Correlation between the total scores pre-/post-STAI in the RP group and pre-/post-STAI in the VP group.

	N	Correlation	Sig.
Pre-/post-STAI (RP)	14	0.458	0.099
Pre-/post-STAI (VP)	16	0.557	0.025

**Table 10:** Mean and standard deviation of the average score pre-/post-STAI in the VP and RP groups for first-timers and non-first-timers.

	MRI (First-Timers/ Non-First-Timers)	N	Mean	Std. Deviation	Std. Error Mean
Pre-STAI (RP)	First	5	17.8000	2.16795	0.96954
	Non-first	9	13.3333	4.38748	1.46249
Post-STAI (RP)	First	5	15.2000	1.92354	0.86023
	Non-first	9	11.4444	2.55495	0.85165
Pre-STAI (VP)	First	4	15.6000	3.20936	1.43527
	Non-first	12	14.8889	2.97676	0.99225
Post-STAI (VP)	First	4	11.2000	1.92354	0.86023
	Non-first	12	11.3333	3.53553	1.17851

**Table 11:** Correlation between different types of preparation (VP and RP) and patient self-satisfaction.

		Levene’s Test for Equality of Variances		*t*-Test for Equality of Means			
		F	Sig.	t	df	Sig. (2-Tailed)	Mean Difference
Total	RP	3.074	0.090	−2.797	28	0.009	−1.98214
	VP			−2.704	20.379	0.014	−1.98214

## Data Availability

All data generated or analyzed during this study are included in this article.

## Abbreviations

CT	Computed Tomography
KAAUH	King Abdullah bin Abdul-Aziz University Hospital
MRI	Magnetic Resonance Imaging
PACS	Picture Archiving and Communication System
QR	Quick Response
RP	Routine Preparation
STAI	State-Trait Anxiety Inventory
VP	Video Preparation
